# Bats Keep Their Ear on the Ball

**DOI:** 10.1371/journal.pbio.0040152

**Published:** 2006-04-18

**Authors:** Françoise Chanut

Imagine you are late for your train. As you approach within sight of the station, the last car pulls off to the left. So you start running in a diagonal to catch up with it. The difficulty is deciding how much to bear left. A different angle will be needed depending on both your speed and the train's speed. For a given set of speeds, mathematics dictates that there is only one bearing angle that will put you on that train.

Determining that optimal bearing and staying the course are the essence of constant bearing (CB), a strategy that sailors, ballplayers, and animals on the prowl use to intercept (or avoid) moving targets. CB is the optimal strategy as long as the target follows a predictable course. Using mental projections of the target's and their own trajectories, a fish pursuing sinking bait and an outfielder tracking a fly ball compute their optimal bearing and move in a straight line toward the point of intercept, adjusting their course along the way if necessary. With targets that change speed and direction unpredictably, a pursuer may still use CB successfully over the stable segments of the trajectory, which is how dogs catch swerving Frisbees. But erratic targets, which change speed and direction quickly and randomly, may not leave the pursuer enough time for CB computations.

Zeroing in on erratic targets is a matter of survival for the big brown bat, which must snag fitfully flying insects before they return to the safety of foliage. Still, a second or two is all a bat needs to locate and catch a straying beetle on the wing, suggesting bats have an efficient way of tracking their prey's erratic motions. Kaushik Ghose, Cynthia Moss, and colleagues observed big brown bats capturing flying insects in a laboratory setting. They report in a new study that, rather than CB, the bats rely on constant absolute target direction (CATD), a strategy that can be shown mathematically to be most time efficient for tracking erratic motions and is similar to that used by search missiles to latch onto moving targets.

Big brown bats use sound rather than light to sense their surroundings. They emit short ultrasonic shrieks into the air and listen to the returning echoes to detect objects around them, quickly adjusting their flight to approach an insect or avoid an obstacle. The researchers released a big brown bat and a flying insect in a large dark room. Using high-speed infrared cameras and a battery of microphones, they recorded the flight path of bats and insects, as well as the bats' shrieks and echoes. Computer analyses of the recordings allowed them to measure the speed and direction of the flights, and calculate a theoretical optimal bearing for each point on the bat's path. From the direction of the shrieks and echoes, they could deduce the orientation of the bat's head relative to the insect, a situation equivalent to detecting the gaze of visual animals.

They found that after spending some time locating the insect, the bats quickly adopted the same flight path as would be predicted if they continuously recalculated bearing angles to match their prey's erratic trajectory. Rather than concluding that bats are geometry wizards, the researchers propose that they use a simple trick to cheat on the math.

One of the geometrical properties of the bat's flight path is that theoretical lines drawn from the bat to the insect would appear as a series of parallels to an external observer (hence the term CATD). Previous research has shown that the big brown bat locks its head direction to the target position—continuously looking at the target during pursuit, like a baseball player keeping his eye on the ball. The researchers suggest that when adopting the CATD strategy with its head locked to the target, all the bat has to do to simplify the math is to ensure its head does not rotate in space as its body swoops and glides to follow its prey.

In fact, the bat may possess a reflex similar to the one that links sensations of imbalance in our inner ear to a posture adjustment. This reflex would allow the bat to quickly correct any deviation of its body's alignment with the prey, thus insuring its unerring aim.

## 

**Figure:**
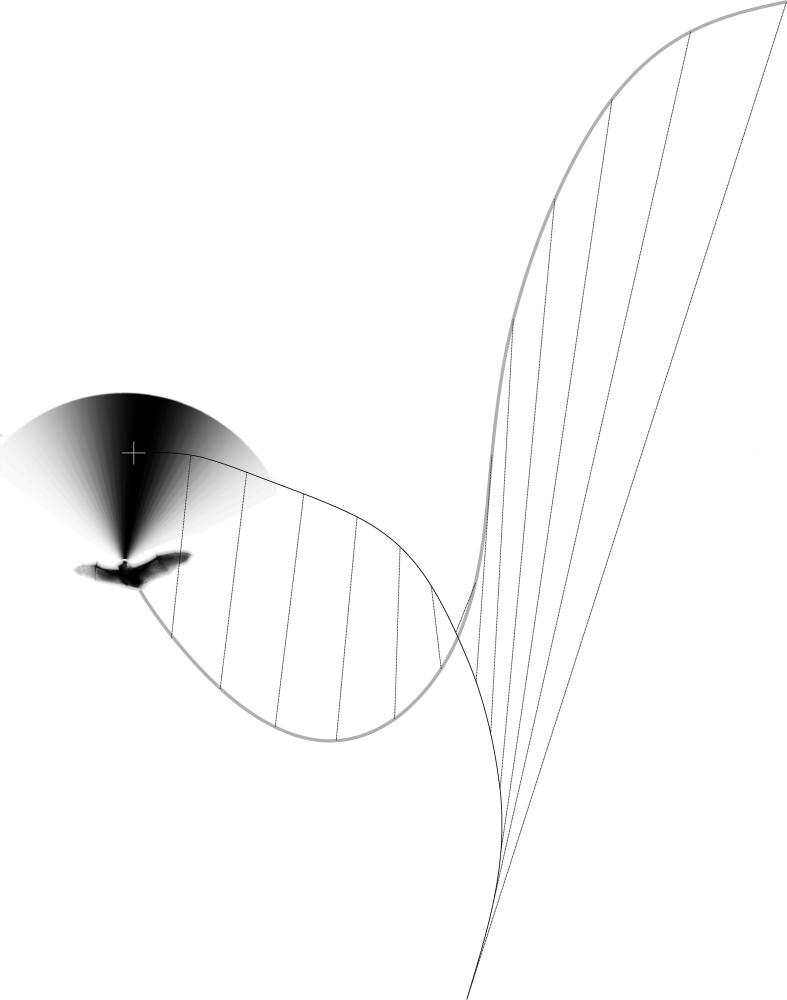
An echolocating bat pursues a fast and erratically moving insect (white cross, black line) by keeping the absolute direction to the target a constant (bearing lines are parallel) while maintaining a lock on the target with its sonar beam (grayscale fan pattern).

